# Dachshund and C-terminal Binding Protein bind directly during *Drosophila* eye development

**DOI:** 10.17912/micropub.biology.001106

**Published:** 2024-03-10

**Authors:** Surya Jyoti Banerjee, Jennifer Curtiss

**Affiliations:** 1 Biological Sciences, Texas Tech University, Lubbock, Texas, United States; 2 Biology, New Mexico State University, Las Cruces, New Mexico, United States

## Abstract

The transcription factor Dachshund (Dac) and the transcriptional co-regulator C-terminal Binding Protein (CtBP) were identified as the retinal determination factors during *Drosophila* eye development *.* A previous study established that Dac and CtBP interact genetically during eye development. Co-immunoprecipitation assays suggested that both molecules interact in the *Drosophila* larval eye-antennal disc. Our present study shows that Dac and CtBP bind each other directly, as determined by GST pull-down assays. Thus, our results demonstrate the molecular mechanism of Dac and CtBP interaction and suggest the direct binding of these two transcription regulators in the cells of the eye disc promotes the *Drosophila* eye specification.

**Figure 1. GST pulldown assays showing CtBP and Dac bind directly in vitro.**
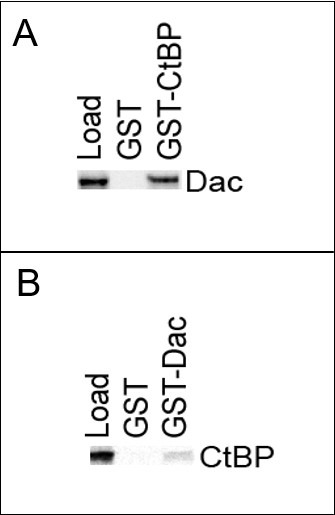
**Figure 1. Panel A** shows that the GST-CtBP but not the GST protein can pull down the Dac protein. The Load in the first lane contains the 10% sample from the in vitro translated Dac protein, and the protein band in the third lane corresponds to the Dac which is pulled down by the GST-CtBP. The absence of the same band in the middle lane indicates that only GST cannot pulldown Dac, suggesting CtBP binds and pulldowns the Dac *in vitro* . **Panel B** shows that the GST-Dac but not the GST protein can pull down the CtBP protein. The Load in the first lane contains the 10% sample from the in vitro translated CtBP protein, and the protein band in the third lane corresponds to the CtBP which is pulled down by the GST-Dac. The absence of the same band in the middle lane indicates that only GST cannot pulldown CtBP, suggesting Dac binds and pulldowns the CtBP *in vitro* .

## Description

The larva of the fruit fly *Drosophila* contains a pair of eye-antennal discs that are the precursors of the two adult compound eyes as well as other head structures. The eye disc is made of two layers; an overlying squamous epithelium layer called peripodial epithelium, and a columnar layer called the disc proper that produces the adult eyes (Bessa and Casares 2005; Kenyon et al. 2003; Moses 2002; Treisman 2013) . The cells in the eye disc undergo proliferation up to the second instar larval stage. During the third larval instar, a dorsal-ventral groove called the morphogenetic furrow initiates at the posterior margin of the eye disc proper and progresses from the posterior to the anterior of the disc proper. The cells ahead of the furrow continue to divide until late in larval development, cells immediately anterior to the furrow are committed to become retinal tissue, and differentiated photoreceptors are established posterior to the furrow (Carthew 2007; Curtiss and Mlodzik 2000; Lebovitz and Ready 1986; Wolff and Ready 1991) .

The fate of the eye precursor cells in the *Drosophila* larval eye discs depends on the precise interactions between transcription regulatory proteins, the players of a complex signaling pathway, popularly known as the retinal determination network. These interactions spatiotemporally regulate differential expression of the *Drosophila* retinal determination genes most of which exhibit conserved function in mice and humans (Baker and Firth 2011; Desplan 1997; Kumar 2010; Silver and Rebay 2005; Weasner et al. 2004) . Therefore, it is very important to understand the mechanism of such molecular interactions, especially for treating human congenital eye diseases like aniridia and blindness.

For instance, the following three transcription regulators in the retinal determination network have been shown to play important roles in the *Drosophila* eye development through their interaction with each other. The *Drosophila* ortholog of human PAX6 is Eyeless (Ey). It is the master transcription factor of the retinal determination network. Loss of Ey function results in smaller eye discs, and very small or no adult eye phenotype (Bessa et al. 2002; Clements et al. 2009; Czerny et al. 1999; Halder et al. 1998; Halder et al. 1995; Hoang et al. 2010; Quiring et al. 1994) . A null mutation in another conserved nuclear protein, the Dachshund (Dac), results in the absence of the morphogenetic furrow in the *Drosophila* larval eye discs, and lack of the adult eyes (Anderson et al. 2006; Caubit et al. 1999; Hoang et al. 2010; Mardon et al. 1994; Pappu et al. 2005) . Conversely, ectopic expression of ey or dac can produce ectopic eyes (Chen et al. 1997; Halder et al. 1995; Shen and Mardon 1997) suggesting that these molecules are hierarchic in the retinal determination network. Evidence suggests that Ey promotes both cell proliferation and retinal cell-fate determination (Bessa et al. 2002; Jiao et al. 2001; Kronhamn et al. 2002; Weasner et al. 2009) , and Dac limits proliferation but promotes retinal fate determination in the eye precursor cells at an appropriate speed (Brás-Pereira et al. 2015; Brás-Pereira et al. 2016). A conserved transcriptional co-regulator C-terminal Binding Protein (CtBP), known for connecting chromatin remodeling molecules with transcription factors, also regulates the fly eye development (Bhambhani et al. 2011; Chinnadurai 2002; 2003; 2007; 2009; Datta et al. 2011; Kumar et al. 2002; Kuppuswamy et al. 2008; Nibu et al. 1998; Phippen et al. 2000; Shi et al. 2003; Turner and Crossley 2001; Zhang and Arnosti 2011) . Loss of function mutations in CtBP increases cell proliferation anterior to the furrow, whereas over-expression of CtBP by *ey-Gal4* driver produces smaller adult eyes; these results suggest that CtBP limits the proliferation of eye progenitor cells and regulates their differentiation in the eye disc (Eusebio et al. 2018; Hoang et al. 2010) .

Interestingly, the expressions of Ey, Dac, and CtBP overlap in front of the morphological furrow in the *Drosophila* larval eye disc (Bessa et al. 2002; Phippen et al. 2000; Poortinga et al. 1998) . Reflecting on their roles in the eye development and expression pattern in the eye disc, it is not a surprise that these molecules can interact with each other and thereby control the fruit fly eye development. Accordingly, Hoang et al. (2010) showed that the small eye phenotype created by *ey-Gal4* -induced upregulation of *ey* ( *ey>ey* ) was dominantly suppressed by a *ctbp* null allele. Similarly, the small eye phenotype in the CtBP over-expressing ( *ey>CtBP* ) flies was dominantly suppressed by a hypomorphic allele of *ey.* Additionally, the smaller adult eye phenotype in *ey>CtBP* flies was dominantly suppressed by a *dac* null allele, suggesting that Ey and CtBP, and CtBP and Dac interact genetically during eye development. To further dissect the mechanism of such interaction, it was shown that Dac and Ey (Hoang et al. 2010; Okamoto et al. 2012) , and Ey and CtBP were present in co-immunoprecipitated products isolated from eye-antennal disc lysates suggesting that these molecules interact with each other in a complex (Hoang et al., 2010) . However, Hoang et al. (2010) were unable to demonstrate direct binding between Ey and CtBP by GST pull-down assay suggesting that another molecule links them within the molecular complex in the cells of *Drosophila* larval eye disc. In this scenario, we predicted that Dac could be the potential mediator molecule to link Ey and CtBP due to another fact that Dac contains a PRDLS motif (amino acid residues 491 - 495) which was known to be critical for CtBP binding by its other partners (Chinnadurai 2007; Hoang et al. 2010; Zhao et al. 2007) . We therefore hypothesized that Dac could directly bind with CtBP and could potentially link CtBP with Ey in a complex during eye development.

To test this hypothesis in *vitro* , we performed GST (Glutathione S-Transferase) pull-down assays *.* We cloned GST, GST-Dac, and GST-CtBP coding DNA sequences (those encoding the different “Bait” proteins) separately in the pGex 4T-2 expression vector and induced each protein expression by 0.5 mM IPTG in BL21 *E. coli* cells followed by protein purification from the cell lysate. We separately expressed biotinylated Dac or CtBP proteins (the “Prey” proteins) from their coding DNA sequence (CDS) cloned in the pTNT vector through *in vitro* transcription and translation (IVT) (please check methods for the detail protocols). We confirmed the expression and successful purification of these proteins by observing strong bands of proteins corresponding to their molecular weights by Coomassie-Blue staining of SDS-PAGE protein gels, and by Western Blot with anti-GST antibody used for Bait proteins and HRP-Streptavidin for the biotinylated Prey proteins.

To perform the pull-downs, we mixed purified GST or GST-Dac proteins with magnetic-glutathione beads to pulldown the biotinylated CtBP protein. Conversely, we mixed the GST or GST-CtBP proteins with magnetic-glutathione beads to pulldown the biotinylated Dac protein. We observed that the GST-Dac but not the GST could pull down CtBP, and GST-CtBP but not GST could pull down Dac in two independent repeat pulldown experiments. We concluded that CtBP and Dac can bind directly with each other. This result was consistent with the idea that Dac played the role of a mediator molecule for the interaction between CtBP and Ey during eye development.

In the future, it will be interesting to investigate whether the PRDLS motif in Dac is responsible for its binding with CtBP, and if both CtBP and Dac can be found in a complex immunoprecipitated by Ey from a tissue lysate prepared from the larval eye-antennal discs. This information can be useful to find their target genes, different molecular partners in complexes that regulate the process of the target gene expression, and the mode of gene expression regulation. Most importantly, this information can aid our current understanding of the impact of such gene regulation on cell behavior in the context of organ development.

## Methods

We used the MagneGST Protein Purification System with Glutathione beads (Promega 17316) to purify the GST or GST-fused Bait proteins and to perform the GST pulldown assays. We used the TNT t7 quick coupled *in vitro* transcription and translation (IVT) system (Promega L1170) for producing the biotinylated Prey proteins by *in vitro* transcription and translation (IVT). In both cases, we followed the manufacturer’s protocol with the changes described below. The pGEX 4T-2 vector was used to produce GST (Glutathione S-Transferase) and GST fusion Bait (GST-CtBP and GST-Dac) proteins. The pTNT vector was used to produce the Prey proteins by *in vitro* transcription and translation. Different combinations of the Bait and Prey proteins were used in the pull-downs (table 1).

**Table 1. Bait and Prey protein combinations used in the GST pulldown assays.**

**Table:** 

**GST-pulldown combinations**	**Bait Protein**	**Prey Protein**
**A. Experimental combination to test if the GST-CtBP but not the GST only (control) can pull down Dac protein.**	**GST-CtBP**	**Dac**
	**GST**	**Dac**
**B. Experimental combination to test if the GST-Dac but not the GST only (control) can pull down CtBP protein.**	**GST-Dac**	**CtBP**
	**GST**	**CtBP**

**Table 1:** The Bait proteins were expressed in the BL21 cells transformed with the GST-tagged coding regions of above listed genes cloned under the *tac* promoter in pGEX 4T-2 plasmid. The Prey proteins were expressed using an *in vitro* translation system using the pTNT plasmid containing the coding sequence of the above-listed genes cloned under the T7 promoter in the plasmid.

To express proteins, first, we dissected 80 eye-antennal discs from the third instar wild-type larvae in ice-cold 1X PBS (10X Phosphate Buffer Saline consists of 1.37 M NaCl, 27 mM KCl, 18 mM KH _2_ PO _4,_ and 100 mM Na _2_ HPO _4_ in 1 Liter of distilled water). We isolated total RNA from eye-antennal discs using the Qiagen RNeasy kit (Qiagen 74004) following the manufacturer's protocol. 1 µg of RNA was used to make cDNA using the iScript Reverse Transcriptase Supermix (BioRad 1708841) kit following the manufacturer's protocol. The cDNA was used as a template to amplify CtBP and Dac coding DNA sequences (CDS) using PCR. PCR products were cloned into pGEX 4T-2 or pTNT plasmids by In-Fusion cloning (In-Fusion HD Cloning Plus CE, Takara Bio/ ClonTech 638916), and were verified by sequencing. The pGEX 4T-2 plasmids that contained either the GST, GST-Dac, or GST-CtBP CDS that is the “Bait” protein-coding sequences were transformed into the BL21 cells (NEB-C2530H from New England Biolabs) to express the GST or GST-fused Bait proteins. GST and GST-fused protein production was induced by adding 0.5 mM IPTG (Sigma I6758) in the Luria Broth media for 4 hours at 28 ^o^ C on an incubator shaker at 250 RPM. Then the proteins were purified in 100 µl Bugbuster (Invitrogen/ Thermo Scientific B-PER 78243) by lysing the BL21 cells expressing a specific Bait protein. In this lysis reagent, 1mM PMSF (Sigma P7626), 1X Protease inhibitor cocktail (Pierce/ Thermo Scientific- 88666), and 1 µl of DNase (Invitrogen/ Life Technologies 18068-015) were added. Whereas, pTNT plasmids with either the Dac or CtBP CDS that is the “Prey” protein-coding DNA sequences cloned in them were transformed into the specific *E. coli* (NEB-C3019H from New England Biolabs) cells for plasmid amplification and isolation. The pTNT-Dac or pTNT-CtBP plasmids were purified using a column-based kit (Qiagen 27106). The Dac or CtBP “Prey” proteins were separately synthesized *in vitro* using the purified plasmids and Promega’s IVT kit. IVT proteins contain lysine residues biotinylated at the ε-amino group. We did Western Blots to verify and confirm the expression of the GST (~26 kDa protein), GST-Dac, and GST-CtBP proteins using an anti-GST antibody (MA4-004 from Thermo Scientific) and HRP conjugated secondary antibody. Additionally, we did Western Blots to verify and confirm the expression of the Dac (~119 kDa protein) and CtBP (~42.5 kDa) *in vitro* translated proteins using Peroxidase-Streptavidin (016-030-084 from Jackson Immuno) antibody.

To perform the pulldown assays, aliquots of washed and equilibrated Glutathione beads were resuspended in the 20 µl GST wash buffer. One aliquot of the beads was incubated with 20 µl of the GST-fused Bait proteins (GST-Dac or GST-CtBP) and the other aliquot with 20 µl of GST protein (negative control) for 30 min at room temperature (RT) and washed three times with 500 µl of GST wash buffer per wash. The treatment enabled GST or GST-fused proteins (GST-Dac or GST-CtBP) to bind with the Glutathione beads. Next, 20 µl of the *in vitro* synthesized Prey protein was added to the Glutathione beads plus GST or Glutathione beads plus GST-fused proteins in microfuge tubes and incubated for 1 hour at RT with gentle rocking. The different Bait and Prey protein combinations incubated together are shown in Table 1. The incubated samples were washed three times with 500 µl of GST wash buffer, and mixed with 20 µl protein denaturing solution [2x Laemmli Sample Buffer (BioRad 1610737) and Beta-mercaptoethanol (Sigma 444203) mixed in 19:1 ratio] in microfuge tubes and heated at 65 ^o^ C for 1 minute. Following that, the samples were run on an 8% SDS-PAGE denaturing gel and transferred to PVDF membranes. The membranes were developed by Western Blot using the Peroxidase-Streptavidin (016-030-084 from Jackson Immuno) and ECL-based buffers to detect the presence of the Prey ( *in vitro* translated and biotinylated Dac or CtBP) proteins precipitated by the GST or GST-fused Bait (GST-CtBP or GST-Dac) proteins (table 1).

## Reagents

TNT t7 quick coupled in vitro transcription/translation (IVT) system (Promega L1170), Glutathione beads (Promega 17316), PBS, Qiagen RNeasy kit (Qiagen 74004), iScript Reverse Transcriptase Supermix (BioRad 1708841), In-Fusion HD Cloning Plus CE, Takara Bio/ ClonTech 638916, BL21 cells (NEB-C2530H), IPTG (Sigma I6758), Bugbuster (Invitrogen/ Thermo Scientific B-PER 78243), PMSF (Sigma P7626), Protease inhibitor cocktail (Pierce/ Thermo Scientific- 88666), DNase (Invitrogen/ Life Technologies 18068-015), E. coli (NEB-C3019H), Plasmid miniprep kit (Qiagen 27106), Peroxidase-Streptavidin (016-030-084 from Jackson Immuno), anti-GST antibody (MA4-004 from Thermo Scientific), etc.
